# Venous Thromboembolism Prophylaxis in Maxillofacial Surgery

**DOI:** 10.7759/cureus.70077

**Published:** 2024-09-24

**Authors:** Inês Camelo, Flávia Pereira, Mariana Cebotari, Manuel Sousa, Lígia Coelho

**Affiliations:** 1 Maxillofacial Surgery Department, Centro Hospitalar Universitário de São João, Porto, PRT

**Keywords:** caprini score, maxillofacial surgery, pharmacological prophylaxis, venous thromboembolism, venous thromboembolism prophylaxis

## Abstract

The aim of this article is to conduct a literature review concerning the need for venous thromboembolism (VTE) prophylaxis among patients undergoing maxillofacial surgery. This article addresses the diagnosis of VTE, patient risk stratification, and prophylactic measures suitable for the various groups of interest in maxillofacial surgery. From a pool of 44 articles identified, nine were chosen as the most pertinent to the aims of this review. Despite limited data on this topic, individuals undergoing oral and maxillofacial surgery generally exhibit a low risk of VTE. This review emphasizes the importance of implementing prophylactic measures tailored to individual risk factors. Pharmacological prophylaxis merits consideration, particularly when the Caprini score is ≥7. However, further research is needed to optimize prophylactic strategies in maxillofacial surgery.

## Introduction and background

Venous thromboembolism (VTE) poses a significant challenge in hospitalized individuals, with deep vein thrombosis (DVT) and pulmonary embolism (PE) being prevalent complications [1-6]. VTE phenomena are the leading cause of preventable death in perioperative patients. In addition, the morbidity associated with these events is significant, with 5% of PE cases developing cardiac dysfunction and 20-50% of DVT cases developing post-thrombotic syndrome. Therefore, early diagnosis, prevention, and treatment of VTE events are crucial [3]. This literature review delves into the diagnosis, risk stratification, and prophylaxis of VTE events, particularly in the context of maxillofacial surgery. Considering the growing number of patients with increasingly complex comorbidities, along with the high frequency of oncologic and reconstructive surgeries among the population undergoing maxillofacial surgery, the limited number of studies and reviews on this matter is concerning. This article intends to serve as a warning to all maxillofacial surgery specialists regarding the importance of VTE prophylaxis in their patient population.

## Review

Methods

A literature review was done using the PubMed and ScienceDirect databases. A total of 44 articles were identified based on the following search terms: "thromboembolism prophylaxis," "venous thromboembolism prophylaxis," "thrombosis," and "maxillofacial surgery." Duplicate articles, non-English articles, and those deemed unsuitable, after reviewing the title and abstract, were excluded. Ultimately, 23 articles were selected for a critical review. Given the lack of literary references on the subject, it was decided to include case reports and non-original articles, such as systematic reviews and literature reviews. Following a comprehensive review, nine articles were considered the most pertinent as they aligned closely with the theme and objectives of this paper.

Diagnosis

VTE encompasses DVT, PE, or a combination of both. It stands as a prevalent complication among hospitalized individuals, with approximately 900,000 cases reported annually [1,2]. PE stemming from DVT stands as the primary cause of preventable hospital mortality [3]. Acknowledging Virchow's triad is crucial in understanding thrombogenesis, which underscores venous stasis, endothelial injury, and hypercoagulability as pivotal factors. Surgical patients are particularly susceptible to VTE due to an array of acquired or genetic risk factors that can amplify the occurrence of these triadic components [1]. While a significant portion (30% to 50%) of DVT cases may exhibit no symptoms, recognizing clinical indicators is paramount for diagnosis. Observable signs encompass unilateral lower limb swelling, pain, palpable hard veins, redness of the skin, tenderness, and loss of ipsilateral peripheral pulses (potentially present with extensive DVT). Progression to PE can manifest as chest pain, dyspnea, decreased oxygen saturation, tachypnea, tachycardia, and hemoptysis [1,4]. Doppler ultrasound serves as the primary diagnostic tool for DVT detection. D-dimers can aid in diagnosis as well. However, elevated D-dimer levels may arise not only in DVT but also in conditions like inflammation, pregnancy, surgery, trauma, and hemorrhage. Nevertheless, a negative D-dimer test can effectively rule out DVT or PE, whereas a positive result does not definitively confirm VTE presence [1]. 

Risk stratification

The risk of thromboembolic events varies among patients, with hospitalization ranking among the most significant risk factors for VTE. Hospital stays constitute a unique period where numerous predisposing factors may converge (e.g., surgery, trauma, intravenous catheters, immobility, pregnancy, and chronic conditions). VTE incidence among hospitalized surgical patients can surge up to 150 times higher compared to those undergoing same-day procedures [1,2]. Consequently, stratifying patients according to their VTE risk is imperative. The Caprini Risk Assessment Model (RAM) was developed for this purpose, aiming to individually characterize VTE risk based on specific patient factors. This model encompasses 40 VTE risk factors (e.g., age, malignancy, congestive heart failure, BMI > 25), categorizing patients into one of four risk levels [3,5]. This score has been validated for patients undergoing general, urological, and vascular surgery. More recently, it has been validated for the population of plastic and reconstructive surgery patients and in patients of otorhinolaryngology (ENT) - head and neck surgery. In these latter two groups, it was found that a Caprini score greater than 8 is associated with a significantly higher risk of VTE. However, to date, there are no studies validating this score in patients undergoing maxillofacial surgery [3,5].

VTE prophylaxis

It is known that a patient who survives a VTE episode is at greater risk of subsequently developing pulmonary hypertension, recurrent PE, or post-thrombotic syndrome. In order to minimize this risk, a variety of mechanical and pharmacological measures have been used [5]. Mechanical prophylaxis consists of elastic compression stockings, intermittent pneumatic compression devices, and intermittent foot compression devices [6,7]. Based on a meta-analysis [8], it has been found that mechanical compression equipment reduces the risk of DVT by 60%. It is considered that compression equipment is preferable to elastic stockings for two reasons: there is a lower percentage of skin side effects, and compression equipment appears to be more effective in preventing VTE [3,9]. Pharmacological prophylaxis, in the form of heparin (intravenous or subcutaneous) or oral anticoagulants, has been the main form of thromboembolic prophylaxis for the last 35 years [1]. Unfractionated heparin has generally been replaced by low-molecular-weight heparin (e.g., enoxaparin), due to ease of use and longer duration of action (however, is preferred in cases of renal insufficiency)[1]. With regard to oral anticoagulants, the role of direct oral anticoagulants (DOACs) (e.g., rivaroxaban, apixaban) stands out, due to the advantage of avoiding the need for daily injections and monitoring, with the potential for greater patient adherence in the post-discharge period [1].

Regarding prophylaxis initiation timing, maxillofacial surgery/ENT-head and neck surgery studies are lacking. However, a systematic review and meta-analysis [10] in plastic and reconstructive surgery recommends initiating prophylaxis six to eight hours post-surgery and continuing throughout hospitalization. Limited data are available regarding the risk of VTE in patients undergoing maxillofacial surgery; nevertheless, studies indicate a notably low risk, estimated between 0.15% and 1.6% [1]. Van de Perre et al. [11] documented three cases of VTE (two DVT and one PE) among 2,049 patients (0.15%) who did not receive prophylaxis, monitored post-surgery (following maxillofacial procedures - maxillary and mandibular osteotomies). Babu et al. [4] presented two cases of VTE, specifically DVT. One case arose in a patient undergoing reduction and osteosynthesis of a double mandibular fracture (angle and parasymphysis), while the other occurred after surgery for oral submucosal fibrosis. Blackburn et al. [12] identified two cases of symptomatic DVT in 129 patients undergoing orthognathic surgery (1.6%) without pharmacological prophylaxis, during a three-month postoperative follow-up. In a retrospective study of 411 patients (none of whom received prophylaxis for VTE) undergoing orthognathic and pre-prosthetic surgery, Forouzanfar et al. [2] identified two patients (0.5%) who developed non-fatal PEs. However, they do not rule out the possibility that the incidence of VTE may have been underestimated.

To date, no comparative studies have investigated VTE incidence in maxillofacial surgery patients versus those undergoing surgeries in other specialties. However, Cramer et al. [7] established a correlation between the incidence of VTE in patients undergoing plastic and reconstructive surgery and ENT-head and neck surgery, suggesting similarity in risk levels. Consequently, it is inferred that similar conclusions may apply to the maxillofacial surgery field, given overlapping areas of intervention. Moreover, no published prospective studies have evaluated pharmacological prophylaxis efficacy for VTE in oral and maxillofacial surgery patients. Nonetheless, analogous investigations have been conducted in otolaryngology-head and neck surgery. Cramer et al. [7] found no significant difference in VTE incidence between patients who did (1.2%) and did not undergo prophylaxis (1.3%). However, among patients with a Caprini score >7, those who received prophylaxis exhibited lower thrombotic event likelihood (5.3% vs. 10.4%). Pannucci et al. [13] concluded that thromboembolic prophylaxis benefits solely patients with a Caprini score ≥7.

Some specific cases of interest in maxillofacial surgery 

Obstructive Sleep Apnea

Individuals with obstructive sleep apnea (OSA) constitute a risk group due to their predisposition to hypercoagulability, evidenced by elevated fibrinogen levels, reduced fibrinolysis, and heightened platelet activity [1]. The severity of OSA correlates with increased platelet aggregation, which normalizes with continuous positive airway pressure (CPAP) therapy. The mechanism underlying the heightened VTE risk in OSA patients likely involves multiple factors, including obesity and dyslipidemia. Given the potential ineffectiveness of mechanical prophylaxis in obese patients, pharmacological prophylaxis should be considered [1,14-16].

Orthognathic Surgery

Particularly concerning orthognathic surgery, Huhmann et al. [17] observed that patients enter a post-surgery prothrombotic state, characterized by elevated coagulation markers. Consequently, they may benefit from thromboembolic prophylaxis. A systematic review [17] on this matter revealed a VTE incidence rate of 0% (0/1088) in patients receiving preoperative heparin prophylaxis compared to 0.19% (12/6327) in those who did not. Regarding bleeding necessitating return to the operating room, rates were 2/88 (2.27%) in the heparin group and 11/1994 (0.55%) in the non-prophylaxis group. Thus, pharmacological prophylaxis should be considered based on individual risk stratification in orthognathic surgery patients [17,18].

Oncological Patients

Oncological patients face a heightened VTE risk compared to the general population, resulting in increased morbidity, mortality, and healthcare costs. It is estimated that 5% to 20% of oncological patients will develop VTE. Over 50% of thrombotic events occur within the first three months post-cancer diagnosis. Treatment modalities such as surgery, chemotherapy, hormone therapy, and targeted agents like thalidomide and erythrocyte transfusions further elevate VTE risk, particularly in older males and certain carcinomas. It is thought that the risk of VTE in head and neck surgery is lower than in other cancer surgeries, possibly due to superficial dissection and early patient mobilization [19-23]. Guidelines suggest individually assessing thrombotic and hemorrhagic risks in oncological surgical patients. For those with low hemorrhagic risk, pharmacological prophylaxis is favored over mechanical methods. Conversely, in cases of high hemorrhagic risk, mechanical prophylaxis is preferred. Patients with high thrombotic risk (without significant hemorrhagic risk) may benefit from combined mechanical and pharmacological prophylaxis or pharmacological prophylaxis alone. When both thrombotic and hemorrhagic risks are high, mechanical prophylaxis should be used until hemorrhagic risk diminishes, after which pharmacological prophylaxis can be initiated [19,24].

Strengths and limitations of this literature review

We consider this literature review to be highly relevant to the maxillofacial surgery community. Through it, we aim to alert specialists to an issue that is often underestimated in clinical practice. The lack of studies related to this topic reflects, on the one hand, the insufficient importance given to it, and on the other, hinders the development of standardized recommendations and protocols about the subject. The scarcity of studies on this topic required the inclusion of case reports and non-original articles, which inevitably represents a major limitation of this literature review. Therefore, large-scale prospective studies should be conducted regarding the incidence and prophylaxis of VTE in maxillofacial surgery. 

## Conclusions

Thromboembolic events are a pathology that warrants significant attention, given their often asymptomatic presentation and their substantial contribution to high mortality and morbidity rates among hospitalized patients. Aside from oncological patients, individuals undergoing oral and maxillofacial surgery generally exhibit a low risk of VTE. Nonetheless, despite the limited studies evaluating VTE prophylaxis efficacy in this field, assessing the patient's thromboembolic risk, especially in those predisposed to multiple risk factors, would be advantageous. Pharmacological prophylaxis merits consideration, particularly when the Caprini score is ≥7. Special consideration is warranted for oncological patients owing to their prothrombotic condition. However, given the rising number of patients undergoing treatment with progressively complex comorbidities, it is imperative to conduct research that comprehensively assesses the incidence and risk factors for VTE within the population undergoing maxillofacial surgery.
